# Malignant hypertension-induced multiorgan failure and acute limb ischemia in a young adult: A case report

**DOI:** 10.1097/MD.0000000000043400

**Published:** 2025-07-18

**Authors:** Jun-Chang Jeong, Ung Kim, Jong-Hyun Baek, Jong-Il Park

**Affiliations:** a Division of Cardiology, Yeungnam University Medical Center, Daegu, Republic of Korea; b Department of Thoracic and Cardiovascular Surgery, Yeungnam University Medical Center, Yeungnam University College of Medicine, Daegu, Republic of Korea.

**Keywords:** acute limb ischemia, left ventricular thrombus, malignant hypertension, multiorgan failure, percutaneous transluminal angioplasty

## Abstract

**Rationale:**

Malignant hypertension (MHT) is characterized by a systolic blood pressure (BP) of ≥ 180 mm Hg and/or a diastolic BP of ≥120 mm Hg, accompanied by acute target organ damage. While it often results in multiorgan failure, the development of a left ventricular (LV) thrombus and subsequent acute limb ischemia (ALI) is rare.

**Patient concerns:**

A 26-year-old Korean male with untreated hypertension presented with MHT and discoloration of the right lower extremity. The affected limb exhibited classic signs of ALI.

**Diagnoses:**

Computed tomography angiography revealed a right renal infarction and total occlusion of the right distal superficial femoral artery. Echocardiography demonstrated a reduced ejection fraction of 32% and a LV thrombus. Hybrid 18-fluoro-deoxyglucose (FDG) positron emission tomography/magnetic resonance imaging revealed no abnormal FDG uptake or late gadolinium enhancement, ruling out myocardial inflammation, fibrosis, or scarring and confirming MHT as the cause of LV dysfunction and thrombus.

**Interventions:**

The patient underwent percutaneous transluminal angioplasty and Fogarty embolectomy, successfully restoring blood flow. Fasciotomy was performed to manage reperfusion injury.

**Outcomes:**

The symptoms and blood flow in the right lower extremity were restored. Follow-up echocardiography showed an improved ejection fraction of 50% and resolution of the LV thrombus. The patient continued to improve, was discharged, and is currently undergoing rehabilitation therapy.

**Lessons:**

Hypertension should be proactively managed to prevent life-threatening complications. Timely intervention, particularly in cases of severe complications, is essential for optimizing patient outcomes.

## 1. Introduction

The definition of malignant hypertension (MHT) is not uniform, but it is commonly defined as a systolic blood pressure (BP) of ≥180 mm Hg and/or a diastolic BP of ≥120 mm Hg with acute impairment of target organs. MHT is often underdiagnosed or difficult to distinguish from uncontrolled hypertension due to the limited attention it receives. As a result, its prevalence and incidence are not well established, but the overall incidence is estimated to be approximately 2 cases per 100,000 individuals. The exact etiology is not well understood, but the most common cause is known to be nonadherence to usual oral therapy and abrupt withdrawal of medication. The renin-angiotensin system is thought to be particularly involved. MHT requires immediate BP lowering, and the workup should not delay treatment. MHT is known to cause damage to multiple organs, particularly affecting the brain, eyes, heart, large arteries, and kidneys.^[1–3]^ Left ventricular (LV) thrombus formation in MHT is rare, and acute limb ischemia (ALI) resulting from it is also extremely uncommon. However, once ALI occurs, it can lead to severe consequences such as amputation and death.^[4]^ The classification follows the Rutherford classification.^[5,6]^ The treatment approach and its complications are the same as for any other case of ALI. Anticoagulation is the most important aspect, and in case of reversible ALI, revascularization is crucial.^[7]^ Additionally, attention should be paid to reperfusion injury and compartment syndrome, which are common after revascularization. Diagnosis begins with suspicion and is made clinically, with treatment involving fasciotomy as a surgical emergency.^[4]^

## 2. Case presentation

**Figure 1.**
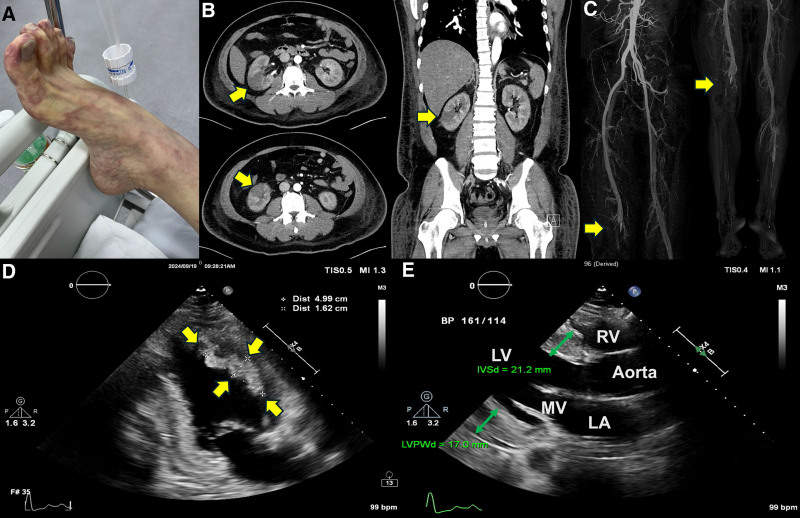
Initial patient condition. (A) Gross appearance, (B) CT angiography showing right renal infarction (arrow), (C) CT angiography showing total occlusion of the right distal superficial femoral artery (arrow), (D) echocardiography showing LV thrombus (arrows), and (E) echocardiography showing LV hypertrophy (green arrows). CT = computed tomography, LV = left ventricle.

**Figure 2.**
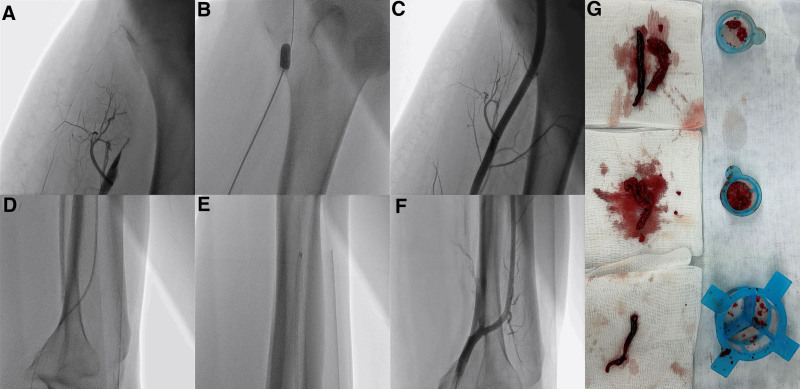
Angiography and procedural images. (A) Total occlusion of the right distal SFA, (B) Fogarty embolectomy, (C) restored blood flow in the right distal SFA, (D) balloon angioplasty below the knee, (E) thrombus aspiration below the knee, (F) restored blood flow below the knee, and (G) removed thrombi. SFA = superficial femoral artery.

**Figure 3.**
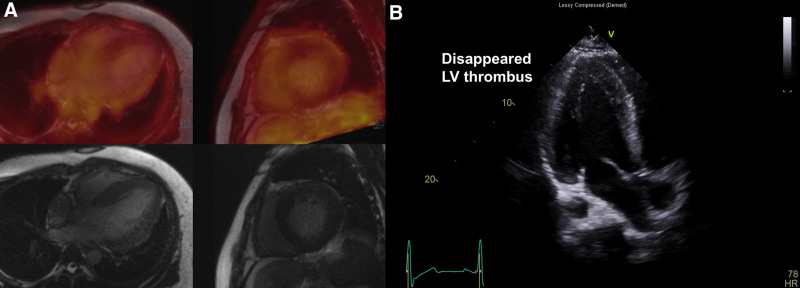
Postprocedural patient evaluation. (A) Hybrid FDG-PET/MR showing no abnormal FDG uptake or LGE, and (B) follow-up echocardiography showing resolution of the LV thrombus. FDG = 18-fluoro-deoxyglucose, LGE = late gadolinium enhancement, LV = left ventricle, PET/MR = positron emission tomography/magnetic resonance.

A 26-year-old male patient with untreated hypertension was transferred to the emergency room from another hospital due to discoloration of the right lower extremity (Fig. 1A). He had been hospitalized for a week due to worsening dyspnea and presented with a hypertensive emergency, with BP over 180/120 mm Hg, a heart rate of 78 bpm, elevated liver enzymes, kidney dysfunction, and a markedly elevated D-dimer level of 20.13 μg/mL. Upon arrival at the emergency room, the right lower extremity exhibited signs of the 6P’s of ALI: pain, pallor, pulselessness, poikilothermia, paresthesia, and paralysis. Computed tomography angiography revealed a right renal infarction (Fig. 1B) and total occlusion of the right distal superficial femoral artery (SFA) (Fig. 1C). Echocardiography revealed a reduced ejection fraction (EF) (32%), concentric LV hypertrophy (interventricular septal thickness of 21.2 mm, posterior wall thickness of 17.0 mm), and a LV thrombus (4.99 × 1.62 cm) (Fig. 1D and E, Supplementary Video 1). Given the presence of LV thrombus and right renal infarction, the cause of ALI was suspected to be embolization of the LV thrombus, followed by thrombus propagation due to activation of the coagulation cascade. Anticoagulation with heparin was initiated immediately, and BP was controlled using intravenous nicardipine. Peripheral angioplasty was performed, revealing occlusion of the distal SFA (Fig. 2A). Percutaneous transluminal angioplasty (PTA) and Fogarty embolectomy were performed with the assistance of a thoracic and cardiovascular surgeon (Fig. 2B). Following Fogarty embolectomy, blood flow in the SFA was restored (Fig. 2C). Subsequently, balloon angioplasty and catheter-based thrombus aspiration were performed from the right anterior tibial artery to the distal part (Fig. 2D and E). After PTA and thrombus aspiration, blood flow below the knee was also restored (Fig. 2F). As a result, multiple thrombi were successfully removed from the right anterior tibial artery to the right SFA using Fogarty embolectomy, balloon angioplasty, and thrombus aspiration (Fig. 2G), and blood flow was restored (Fig. 2C and F). The pulse in the right dorsalis pedis artery was also restored, and all symptoms and signs in the right lower extremity showed improvement. The next day, the patient presented with dark-brown urine, right leg pain, and right leg swelling. Concurrently, laboratory tests showed elevated creatine phosphokinase (29,636 U/L), aspartate aminotransferase (725 IU/L), alanine aminotransferase (886 IU/L), lactate dehydrogenase (1371 IU/L), and serum myoglobin (>1 200 ng/mL). Therefore, under clinical suspicion of reperfusion injury and impending compartment syndrome, an immediate fasciotomy using the double-incision technique was performed. After BP control, the fasciotomy was closed 2 weeks later. Hybrid 18-fluoro-deoxyglucose (FDG) positron emission tomography/magnetic resonance imaging (PET/MRI) showed no abnormal FDG uptake or late gadolinium enhancement (Fig. 3A). These findings indicate the absence of myocardial inflammation, fibrosis, or scaring, confirming that MHT was the cause of the LV dysfunction and thrombus. In addition to MHT and its resulting cardiac dysfunction, no other conventional risk factors for arterial thromboembolism – such as atrial fibrillation, valvular heart disease, aortic atherosclerosis, recent vascular intervention, or known hypercoagulable states – were identified in this patient. Follow-up echocardiography demonstrated an improved EF (50%) and a resolution of the LV thrombus (Fig. 3B, Supplementary Video 2). In addition, hypertensive retinopathy was confirmed following an ophthalmology consultation. Consequently, this case reaffirmed multiorgan involvement due to MHT, affecting the heart, liver, eyes, renal artery, and lower extremities. Finally, all lab findings showed improvement, and multiorgan failure also improved. The patient was discharged with warfarin 2.5 mg once daily, sacubitril/valsartan (Entresto^®^) 200 mg twice daily, carvedilol SR 64 mg once daily, amlodipine 10 mg once daily, and empagliflozin 10 mg once daily.

## 3. Discussion

Hypertension is often perceived as a mild condition, but neglecting treatment or having poor medication adherence can lead to severe consequences, such as MHT. Furthermore, MHT can cause multiple target organ damages and lead to various complications.^[1–3]^ There are few publications on performing PTA for ALI caused by MHT. This is due to its low incidence; however, the treatment approach and complications after revascularization remain the same. In the author’s opinion, the presented case effectively illustrates both the common complication of multiorgan failure and the rare, yet potentially fatal complication of ALI associated with MHT. It is rare for a young patient to develop ALI due to MHT causing reduced LVEF, the formation of an LV thrombus, and its subsequent embolization. Some case reports have described ALI caused by LV thrombus,^[8,9]^ but these cases involved patients with underlying conditions predisposing to coagulopathy. In contrast, our patient was a previously healthy young male, except for untreated hypertension. Hypertension is known to induce a hypercoagulable state.^[10]^ Based on findings from hybrid FDG-PET/MRI, we believe that our patient experienced thrombotic events as a result of MHT. Other common risk factors for arterial thromboembolism-including atrial fibrillation, valvular heart disease, aortic atherosclerosis, malignancy, recent surgery or trauma, and inherited or acquired thrombophilia-were not identified in this case. Additionally, infectious etiologies such as septic emboli from infective endocarditis, which are recognized causes of limb ischemia in young adults, were also considered but found unlikely based on the absence of fever, leukocytosis, or positive blood cultures.^[11,12]^ This absence of alternative prothrombotic conditions further supports MHT as the principal cause of thrombus formation and systemic embolization in this patient.^[13]^ Even if ALI occurs as a result of MHT, its treatment aligns with that of general ALI management. Immediate anticoagulation with heparin is initiated, and early revascularization is attempted based on Rutherford classification if viability is present.^[4–7,9,14]^ The key point is that revascularization should be attempted early while the condition is still reversible. In such cases, even if PTA is performed, it is worth considering how long anticoagulation therapy should be continued. Regrettably, optimal regimens and duration of anticoagulation therapy for LV thrombus and ALI remain anecdotal.^[15]^ In this case, the authors believe that the patient’s favorable clinical outcome was achieved due to early interventions, such as PTA and fasciotomy, with each procedure strategically anticipating the next step. Timely and accurate decision-making, along with prompt intervention, is essential.

## 4. Conclusion

Hypertension should not be considered merely a minor condition or a risk factor for other diseases, but rather as a significant disease in its own right. It is a cornerstone for fatal complications, necessitating early and appropriate treatment. If a fatal complication arises, timely and accurate decision-making alongside prompt intervention can significantly improve patient outcomes. Further evidence on ALI caused by MHT is needed, and analysis of a larger number of cases is essential to guide optimal management.

## Author contributions

**Conceptualization:** Ung Kim, Jong-Il Park.

**Investigation:** Jun-Chang Jeong, Ung Kim, Jong-Hyun Baek, Jong-Il Park.

**Methodology:** Jun-Chang Jeong, Ung Kim, Jong-Hyun Baek, Jong-Il Park.

**Supervision:** Ung Kim, Jong-Il Park.

**Writing – original draft:** Jun-Chang Jeong.

**Writing – review & editing:** Jun-Chang Jeong, Ung Kim, Jong-Il Park.
